# Rose Bengal Conjugated to Lectins for Targeted Antibacterial Photodynamic Treatment

**DOI:** 10.3390/molecules30112381

**Published:** 2025-05-29

**Authors:** Melad Atrash, Iryna Hovor, Marina Nisnevitch, Faina Nakonechny

**Affiliations:** Department of Chemical Engineering, Ariel University, Kiryat-ha-Mada, Ariel 40700, Israel

**Keywords:** conjugation, photosensitizers, Rose Bengal, lectins, concanavalin A, photodynamic antibacterial activity

## Abstract

Due to rising antibiotic resistance, it is necessary to develop alternative ways to combat pathogenic bacteria. One alternative is photodynamic antibacterial chemotherapy (PACT). This work presents the conjugation of the photosensitizer Rose Bengal (RB) to lectins to improve its efficacy against Gram-positive and Gram-negative bacteria. Two lectins, concanavalin A (ConA) and wheat germ agglutinin (WGA), were covalently linked to RB. Spectroscopic and chromatographic data confirmed successful conjugation. Microscopic examination demonstrated that both lectins agglutinate cells of Gram-positive *S. aureus*, including clinical multidrug-resistant MRSA strains, and Gram-negative *E. coli*, *P. aeruginosa*, and *S. paratyphi* B, although ConA showed a more pronounced reaction. Photodynamic assays showed that ConA-RB achieved complete eradication of *S. aureus* at significantly lower concentrations and light doses than free RB or WGA-RB. ConA-RB also exhibited higher efficacy against Gram-negative bacteria compared to free RB at lower concentrations and shorter illumination periods. WGA-RB was less effective, showing preferential activity against *S. aureus*. Our findings suggest that lectin–RB conjugates offer a promising approach for selective antibacterial treatment under illumination.

## 1. Introduction

Gram-positive and Gram-negative bacteria are major human pathogens prevalent in social communities, particularly in hospitals, where they cause a wide range of infections, from minor to life-threatening conditions with high morbidity and mortality [1,2,3]. Gram-positive bacteria are often associated with skin and respiratory tract infections [4,5,6], while Gram-negative bacteria are commonly involved in severe conditions such as sepsis and multidrug-resistant infections due to their robust defense mechanisms [7]. These pathogens pose significant risks to hospital patients, especially those with surgical wounds and indwelling medical devices. In such cases, bacteria may colonize implanted devices, causing local damage or disseminating via the bloodstream to internal organs, potentially leading to serious illness and death [8,9,10].

The increasing resistance of bacteria to antibiotics and the limited treatment options available present significant challenges in managing bacterial infections, as many species have developed mechanisms to survive antibiotic therapy [11,12,13]. Bacterial resistance may be an inherent property of a microorganism or acquired through de novo mutation, frequently involving the acquisition of extrachromosomal DNA from other bacteria [14,15,16]. While antibiotics have been the standard treatment for bacterial infections for decades, the rise of antibiotic-resistant bacteria necessitates alternative therapeutic approaches [17,18,19,20]. One promising alternative is photodynamic antibacterial chemotherapy (PACT), which offers a targeted strategy for bacterial eradication. PACT involves the use of photosensitizers (PSs), molecules that generate reactive oxygen species (ROS) upon illumination with light at an appropriate wavelength [21,22,23,24]. One of the PSs, Rose Bengal (RB), is a halogenated xanthene dye [25,26] widely employed in PACT due to its high antibacterial activity against Gram-positive bacteria and moderate activity against Gram-negative bacteria [27,28,29,30,31,32].

A critical limitation of photosensitizers is their lack of specificity, which can result in biological incompatibility with different cell types. Strategies such as antibody conjugation and liposome encapsulation are commonly employed to mitigate off-target effects. A new and promising approach to enhance the efficiency of photosensitizers involves their conjugation with lectins, a class of carbohydrate-binding proteins, that recognize and attach specifically to sugar moieties on the surfaces of cells and glycoproteins.

Lectins are a diverse group of proteins that selectively bind specific saccharide components of lipopolysaccharides (LPSs). Concanavalin A (ConA), derived from Jack bean (*Canavalia ensiformis*), is among the most extensively studied and utilized lectins [33,34,35]. ConA exhibits a strong binding affinity for mannose and glucose residues in polysaccharides, allowing it to interact with bacterial surface LPSs in varying capacities [36,37]. Another lectin, wheat germ agglutinin (WGA), obtained from wheat germ (*Triticum aestivum*), exhibits a significant binding affinity for N-acetylglucosamine acid in the peptidoglycan layer of Gram-positive bacteria and poly-β-1,6-linked N-acetylglucosamine in both Gram-negative and Gram-positive bacteria [38]. This affinity allows using WGA for targeted drug delivery to bacteria [39].

Conjugating RB with lectins may increase its activity in eradicating Gram-positive and Gram-negative bacterial cells. Such an approach to treating Gram-negative *Escherichia coli* was introduced in 2020 by Cantelli et al. [40], who proposed a novel antimicrobial strategy using a conjugate of ConA and RB, whereas the conjugation of RB to ConA did not alter the lectin’s specific binding properties.

The combination of the bacterial recognition capabilities of ConA with the photodynamic properties of RB significantly enhances the effectiveness of the latter in PACT against Gram-negative bacteria, which are more resistant to photodynamic action due to their complex cell wall structure. During the treatment, ROS, generated under light activation, are concentrated near the target bacteria, minimizing their spread to the surrounding environment and facilitating the targeted photodynamic destruction of bacteria. This approach not only increases the local concentration of RB at the bacterial surface but also reduces the collateral damage to host cells [40,41].

This research aims to explore RB conjugated to two lectins, ConA and WGA, applied to the targeted and effective eradication of Gram-positive and Gram-negative bacteria and compared to free RB, thus highlighting the potential of such conjugates to become a valuable adjunct or an alternative to traditional antibiotic therapies.

## 2. Results and Discussion

### 2.1. Microscopic Analysis of ConA and WGA Binding with Gram-Positive and Gram-Negative Bacteria

**Figure 1:**
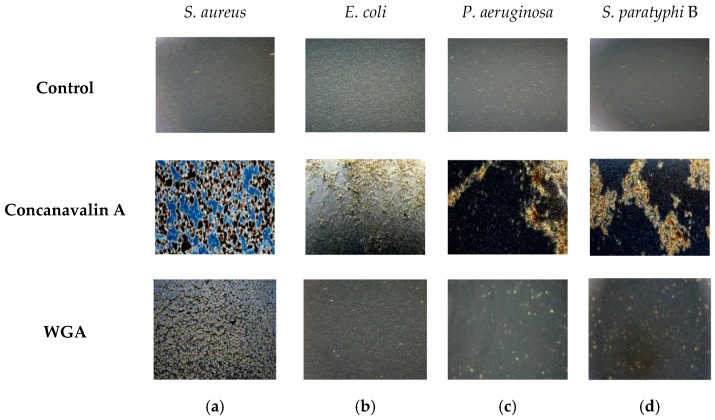
Visualization of interaction between ConA (second line) or WGA (third line) and Gram-positive and Gram-negative bacteria: (**a**) *S. aureus*, (**b**) *E. coli*, (**c**) *P. aeruginosa*, and (**d**) *S. paratyphi* B. The bacterial cells were incubated with ConA for 2 min and immediately examined using an optical microscope.

ConA and WGA are known to bind to bacterial cell walls of Gram-negative and Gram-positive bacteria [39,42,43,44], and at the first stage of our work, this phenomenon was visualized for a number of bacteria. For this purpose, the bacterial cells were incubated with ConA or with WGA for a short period and examined under an optical microscope. The results of this study are presented in Figure 1. The microscopic images show that in all cases, ConA causes a very distinct agglutination of Gram-positive (*Staphylococcus aureus*, Figure 1a) and Gram-negative (*Escherichia coli*, *Pseudomonas aeruginosa*, and *Salmonella paratyphi* B, Figure 1b–d) bacterial cells, whereas control cells remain intact. WGA also agglutinates all the tested bacteria, but a strong effect is seen only in the case of *S. aureus* (Figure 1a), and its binding to Gram-negative cells is less pronounced (Figure 1b–d). These observations enable us to conclude that ConA exhibits a good affinity to all the tested bacteria, especially for the Gram-negative ones, and WCA reacts better with Gram-positive cells than with Gram-negative ones. We assume that both lectins can be used for targeting antibacterial compounds.

### 2.2. Synthesis and Characterization of Lectin–RB Conjugates

The ConA-RB and WGA-RB conjugates were synthesized by attaching the carboxyl group of RB to one of the amino groups of the lectins as described in [40]. The obtained conjugates were separated from the free RB by size exclusion chromatography, where the first fractions eluted in a void volume were conjugates and the second fraction in each case was unbound RB. The number of RB molecules attached to ConA or to WGA was determined by spectral measurements. The ratio of the RB molecules to ConA in the conjugate was found to be 2.8 RB molecules per ConA monomer. In the study of Cantelli et al. [40], it was reported that 2.4 RB molecules were attached to the ConA monomer. In the case of WGA, the number of conjugated RB molecules was 3.1 per WGA monomer. Despite ConA possessing more lysine residues (twelve lysine residual groups, [45]) than WGA (seven lysine residual groups, [46]), the observed similar degree of RB conjugation per monomer can be attributed to differences in lysine accessibility and the three-dimensional structures of the proteins [47]. Not all lysine residues are equally available for conjugation reactions; some may be buried within the protein’s tertiary structure or involved in intramolecular interactions, rendering them less reactive. Additionally, steric hindrance around certain lysine sites can impede the approach of conjugating agents like RB, further limiting effective conjugation. Therefore, the comparable conjugation levels observed are likely a result of these structural and spatial considerations rather than the absolute number of lysine residues.

**Figure 2:**
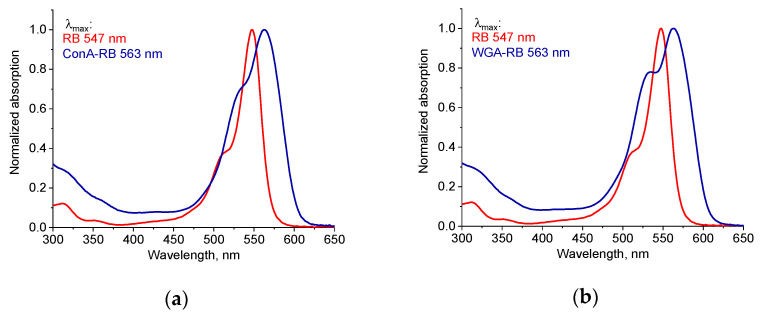
Absorption spectra of free RB (red) and lectin–RB conjugates (blue): ConA-RB (**a**) and WGA-RB (**b**) in 0.1 M PBS, pH 7.4.

The absorption spectra of free RB and the lectin–RB conjugates in the visible region are shown in Figure 2. The absorbance maximum of free RB was observed at 546 nm, which aligns with previously reported literature data [25], whereas the long-wavelength band red-shifted to 563 nm and shorter-wavelength shoulder increased (Figure 2), which clearly indicated the formation of conjugates [48,49]. Similar changes in the RB spectrum due to conjugation with ConA were previously reported by Cantelli et al. [40].

### 2.3. Photodynamic Activity of RB Conjugated with Lectins

The antibacterial photodynamic properties of ConA-RB, WGA-RB, and free RB were tested against Gram-positive and Gram-negative bacteria. The conjugates and free RB were added to suspensions of bacterial cells and the samples were incubated either in the dark or when exposed to the white LED light. In the parallel series, the concentrations of the lectin–RB conjugates and free RB were taken equal relative to RB.

#### 2.3.1. Antibacterial Activity of Lectin–RB Conjugates Against Gram-Positive Bacteria

**Figure 3:**
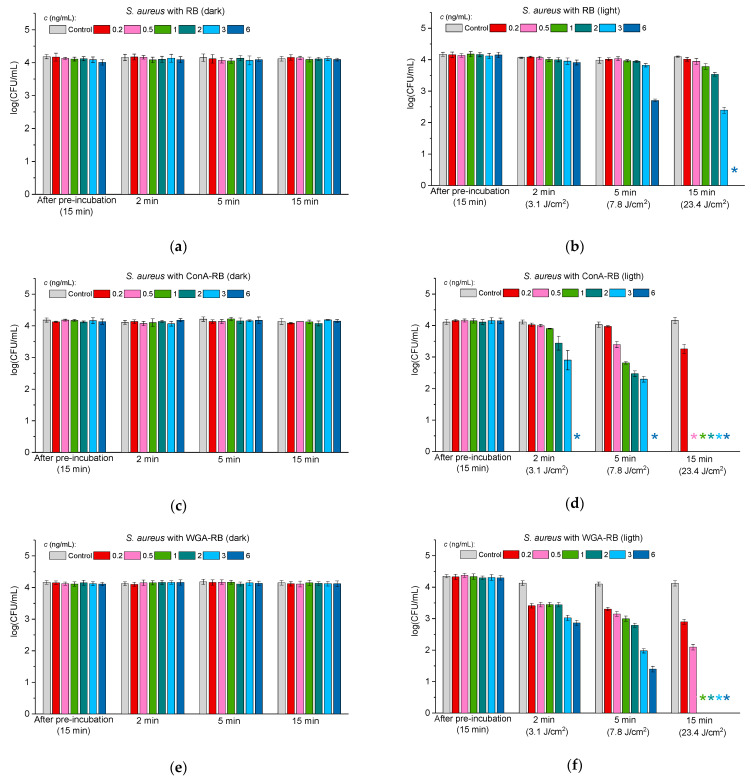
The effect of free RB (**a**,**b**), ConA-RB (**c**,**d**), and WGA-RB (**e**,**f**) on *S. aureus* (MSSA strain) cells incubated in the dark (**a**,**c**,**e**) and under white LED illumination (**b**,**d**,**f**). Error bars represent the STDEV. An asterisk * indicates the total inhibition of bacterial cells.

In the first stage, the activity of free RB was tested against Gram-positive methicillin-susceptible *S. aureus* (MSSA) in the dark (Figure 3a) and under illumination by visible light (Figure 3b). Free RB exhibited no dark toxicity at all the tested concentrations ranging from 0.2 to 6 ng/mL, but under exposure to light, the cells were totally inhibited by RB at the concentration of 6 ng/mL, and partial destruction of cells was achieved within 5 min of illumination (Figure 3b).

As in the case of free RB, neither the ConA-RB nor WGA-RB exhibited any dark toxicity (Figure 3c,e). However, upon exposure to visible light, *S. aureus* was completely eradicated by ConA-RB at the 6 ng/mL concentration already after 2 min (Figure 3d). At lower concentrations of 0.5–3 ng/mL, after 5 min, partial inactivation of cells was observed, and after 15 min, bacteria were eradicated (Figure 3d). WGA-RB was less active than ConA-RB and complete cell eradication was achieved at the concentration of 1 ng/mL only after 15 min of illumination.

For killing cells of *S. aureus* by ConA-RB at the 6 ng/mL concentration it was enough to apply a light dose of 3.1 J/cm^2^. In the case of WGA-RB, the same effect was achieved at 23.4 J/cm^2^ with a concentration of 1 ng/mL, and for free RB at the same light dose of 23.4 J/cm^2^ the killing concentration was 6 ng/mL. The results showed a significant difference between the antibacterial activity of ConA-RB and free RB, with a *p*-value of 0.0368.

These findings suggest that the ConA-RB conjugate is very effective in eradicating Gram-positive bacteria upon light irradiation, even at low concentrations. WGA-RB also showed a significantly higher efficacy than free RB, with a *p*-value of 0.045. The ability of both conjugates to achieve complete bacterial eradication at low concentrations and low light doses allows for their consideration as potent antibacterial agents.

These findings align with previous studies on photodynamic antibacterial therapy, which showed that photosensitizer conjugates enhance bacterial targeting and improve phototoxic efficiency [40,50]. One of the key factors contributing to the enhanced antibacterial efficacy of ConA-RB compared to free RB is its targeted delivery and binding to bacterial cell surfaces. ConA is known to have a high affinity to the external membrane of the bacteria by the selective recognition of LPSs, facilitating the localization of RB at the bacterial membrane [40]. This targeted approach probably enhances ROS generation close to the bacterial membrane, leading to more efficient photodamage. Our previous studies demonstrated that targeted PS delivery significantly increases ROS-mediated bacterial destruction [51]. Furthermore, our results indicate that the ConA-RB conjugate required a significantly lower light dose (3.1 J/cm^2^) to achieve complete bacterial eradication compared to free RB (23.4 J/cm^2^). The increased efficacy at lower light doses suggests that ConA-RB conjugates may be advantageous for clinical applications where minimizing light exposure is desirable to prevent potential cytotoxic effects on host tissues.

#### 2.3.2. Antibacterial Activity Against Gram-Negative Bacteria

Since Gram-negative bacteria are known to be less sensitive to photodynamic treatment than Gram-positive bacteria, it was decided to test the efficiency of ConA-RB against three Gram-negative species: *Escherichia coli*, *Pseudomonas aeruginosa*, and *Salmonella paratyphi* B.

**Figure 4:**
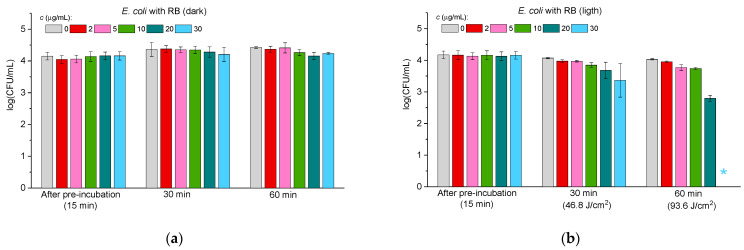

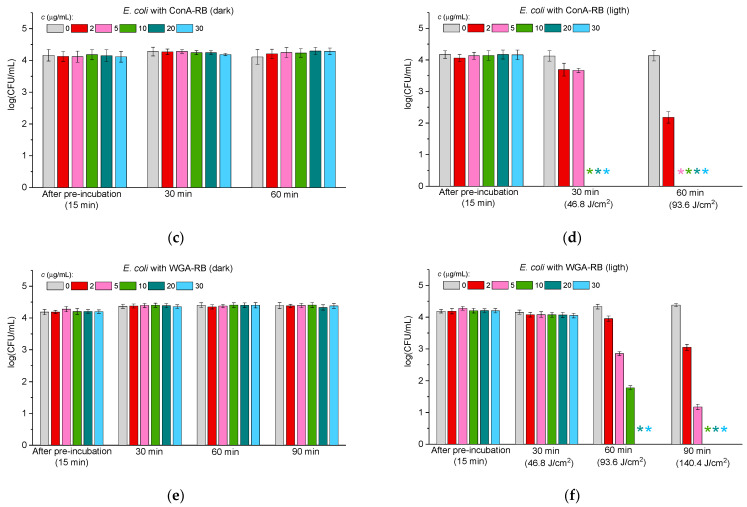
The effect of free RB (**a**,**b**), ConA-RB (**c**,**d**), and WGA-RB (**e**,**f**) on *E. coli* cells incubated under dark conditions (**a**,**c**,**e**) and under white LED illumination (**b**,**d**,**f**). Error bars represent the STDEV. An asterisk * indicates the total inhibition of bacterial cells.

**Figure 5:**
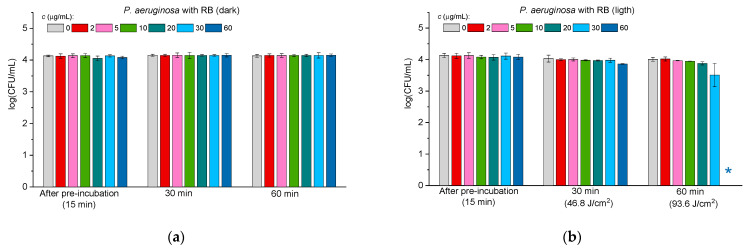

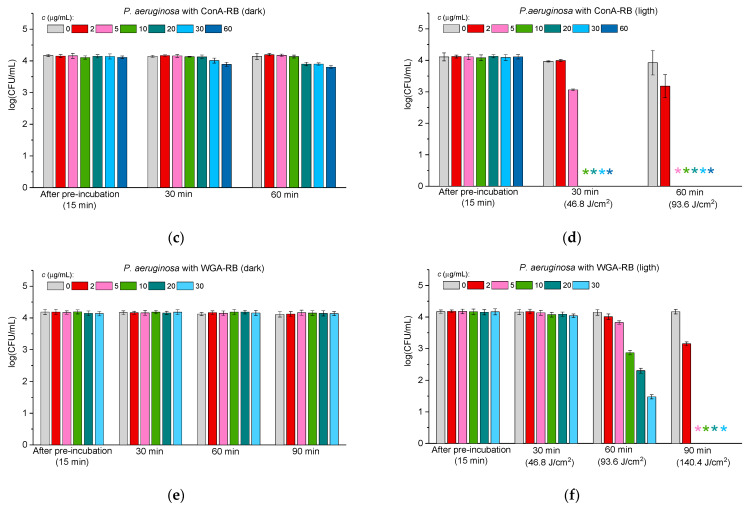
The effect of free RB (**a**,**b**), ConA-RB (**c**,**d**), and WGA-RB (**e**,**f**) on *P. aeruginosa* cells incubated under dark conditions (**a**,**c**,**e**) and under white LED illumination (**b**,**d**,**f**). Error bars represent the STDEV. An asterisk * indicates the total inhibition of bacterial cells.

**Figure 6:**
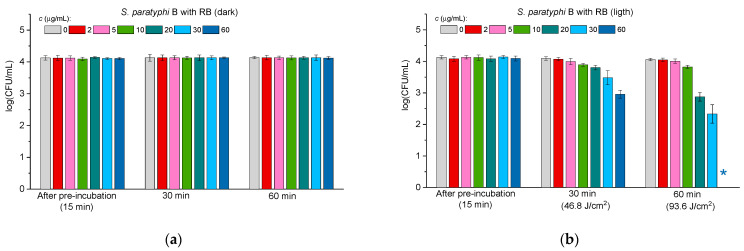

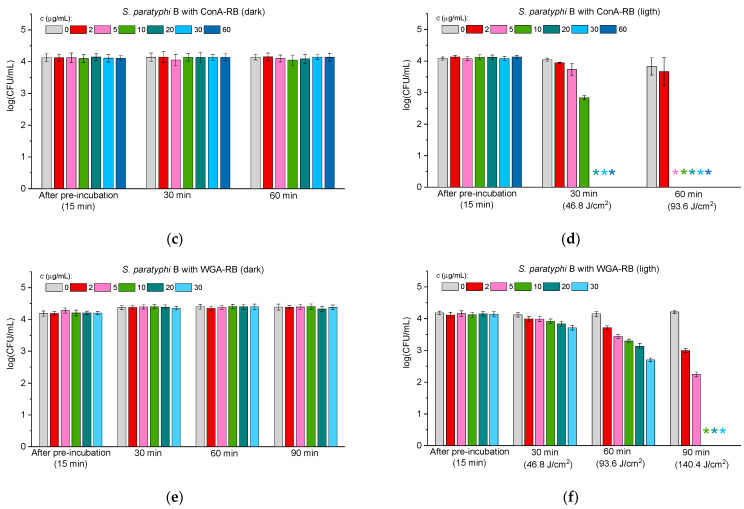
The effect of free RB (**a**,**b**), ConA-RB (**c**,**d**), and WGA-RB (**e**,**f**) on *S. paratyphi* B cells incubated under dark conditions (**a**,**c**,**e**) and under white LED illumination (**b**,**d**,**f**). Error bars represent the STDEV. An asterisk * indicates the total inhibition of bacterial cells.

Figure 4, Figure 5 and Figure 6 illustrate the antibacterial activity against *E. coli* (Figure 4), *P. aeruginosa* (Figure 5), and *S. paratyphi* B (Figure 6) in the case of free RB (panels “a” and “b” in Figure 4, Figure 5 and Figure 6) and the ConA-RB (panels “c” and “d” in Figure 4, Figure 5 and Figure 6) and WGA-RB (panels “e” and “f” in Figure 4, Figure 5 and Figure 6) conjugates at different concentrations under dark incubation and upon visible light illumination.

In the case of all the tested bacteria, free RB exhibited no dark antibacterial toxicity (panels “a” in Figure 4, Figure 5 and Figure 6), but was effective under illumination, killing *E. coli* after 60 min at the concentration of 30 μg/mL (Figure 4b) and *P. aeruginosa* and *S. paratyphi* B at 60 μg/mL (Figure 5b and Figure 6b, respectively). As for free RB, the conjugates ConA-RB and WGA-RB did not show dark activity in any case (panels “c” and “e” in Figure 4, Figure 5 and Figure 6).

Interestingly, the photodynamic antibacterial properties of the conjugates against the group of Gram-negative bacteria were different. ConA-RB, in all cases, was more active than free RB. It eradicated *E. coli* and *P. aeruginosa* after 30 min of illumination at 10 μg/mL (Figure 4d and Figure 5d) and *S. paratyphi* B—after 30 min at 20 μg/mL (Figure 6d). The efficiency of ConA-RB was significantly higher than that of free RB in all cases, with *p*-values of 0.00108, 0.00019, and 0.0161 for *E. coli*, *P. aeruginosa*, and *S. paratyphi* B, respectively. However, WGA-RB was only a little bit more active than RB against *E. coli*, killing it after 60 min at 20 μg/mL (Figure 4f), although this difference was not confirmed statistically (*p*-value = 0.0961). Against *P. aeruginosa* and *S. paratyphi* B, this conjugate was no more active than free RB, with *p*-values of 0.32 and 0.74, respectively. After 1 h of illumination, these cells were not inhibited at all at any concentration and were killed only after 1.5 h of illumination at 5 and 10 μg/mL in the case of *P. aeruginosa* and *S. paratyphi* B, respectively (Figure 5f and Figure 6f).

As in previous studies [52,53,54,55], the results of the current work show that Gram-negative bacteria are more persistent in photodynamic treatment than Gram-positive ones. To eradicate the Gram-positive *S. aureus*, much lower concentrations of free RB and of both conjugates and shorter illumination were needed than for any Gram-negative bacteria (Figure 3, Figure 4, Figure 5 and Figure 6). Among the three Gram-negative species tested, *E. coli* was the most susceptible to photodynamic treatment, followed by *P. aeruginosa*, while *S. paratyphi* B demonstrated the highest persistence to PACT. These findings align with previous reports demonstrating that RB conjugation enhances its photodynamic efficiency by improving bacterial adhesion to *E. coli* cells [40]. As to the cells of *P. aeruginosa*, it was reported previously that they exhibit reduced susceptibility to PACT due to their intrinsic persistence, probably related to efflux pump activity or production of exopolysaccharide alginate [56]. Similarly, *S. paratyphi* B demonstrated higher persistence against both RB conjugates and against free RB. These findings further corroborate the notion that bacterial cell wall composition plays a crucial role in susceptibility to PACT [57].

Our results are consistent with previous findings indicating that the outer membrane of Gram-negative bacteria, rich in lipopolysaccharides, limits PS penetration and subsequent ROS-mediated damage [52,53].

#### 2.3.3. Antibacterial Activity Against Multidrug-Resistant Bacteria

**Figure 7:**
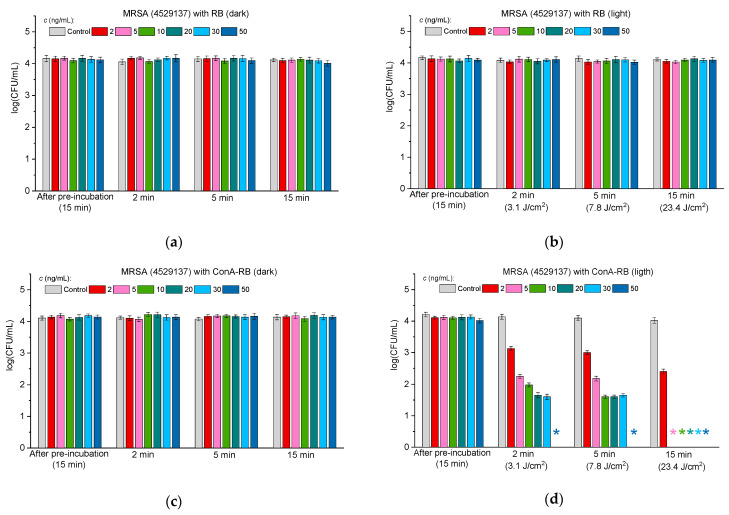

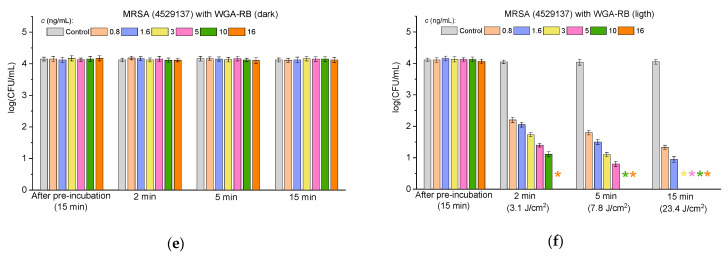
The effect of free RB (**a**,**b**), ConA-RB (**c**,**d**), and WGA-RB (**e**,**f**) on MRSA 4529137 cells incubated under dark conditions (**a**,**c**,**e**) and under white LED illumination (**b**,**d**,**f**). Error bars represent the STDEV. An asterisk * indicates the total inhibition of bacterial cells.

**Figure 8:**
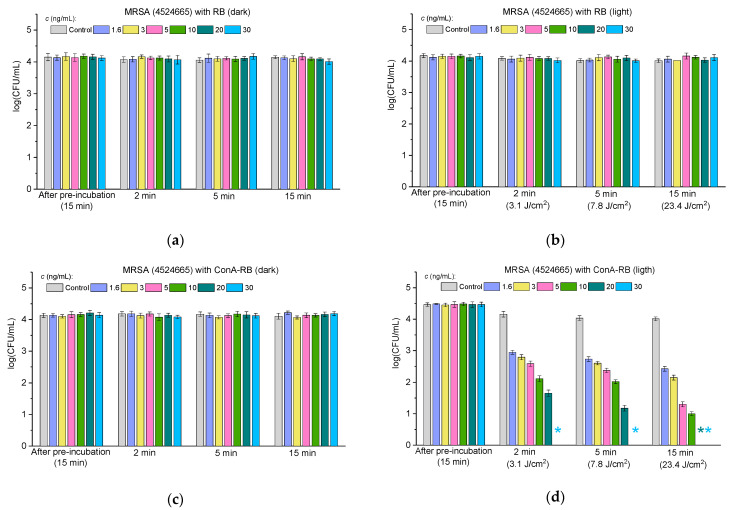

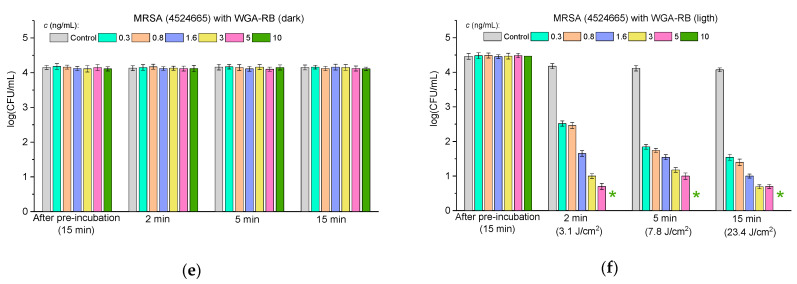
The effect of free RB (**a**,**b**), ConA-RB (**c**,**d**), and WGA-RB (**e**,**f**) on MRSA 4524665 cells incubated under dark conditions (**a**,**c**,**e**) and under white LED illumination (**b**,**d**,**f**). Error bars represent the STDEV. An asterisk * indicates the total inhibition of bacterial cells.

To find out if the RB–lectin conjugates exhibit antibacterial properties against multidrug-resistant bacteria, we evaluated the antibacterial activity of the conjugates against clinical strains MRSA 4524665 and MRSA 4529137. According to the data of clinical characterizations of these strains, the former strain, in addition to methicillin, is resistant to oxacillin and penicillin and the latter strain is resistant to oxacillin, penicillin, erythromycin, clindamycin, gentamycin, and ofloxacin and is intermediate to ceftaroline. Figure 7 and Figure 8 demonstrate the antibacterial activity of free RB (panels “a” and “b” in Figure 7 and Figure 8) and RB–lectin conjugates (ConA-RB in panels “c” and “d” and WGA-RB in panels “e” and “f” in Figure 7 and Figure 8) against the clinical strains MRSA 4529137 (Figure 7) and MRSA 4524665 (Figure 8) at different concentrations under dark incubation and upon visible light illumination.

The findings presented in Figure 7 and Figure 8 demonstrate that RB–lectin conjugates retain their photodynamic antibacterial activity even against multidrug-resistant strains of *Staphylococcus aureus* (MRSA). Both MRSA 4524665 and MRSA 4529137 showed significant susceptibility to the ConA-RB and WGA-RB conjugates upon visible light irradiation, whereas free RB, in contrast to its effect on the MSSA strain, did not affect bacterial viability in the tested range of concentrations (Figure 3). The results obtained with lectin-conjugated RB extend our previous observations with MSSA, reinforcing the hypothesis that lectin conjugation enhances the specificity and potency of photodynamic inactivation.

When comparing the two groups—MSSA and MRSA—one can see that ConA-RB maintains superior antibacterial performance across both strain types, although variations were observed in the concentrations required for total cell inhibition. In the case of MSSA, complete eradication was achieved at 6 ng/mL within 2 min, whereas for MRSA 4524665 and MRSA 4529137 the concentrations were 30 and 50 ng/mL, respectively. Notably, the WGA-RB, which was less potent than ConA-RB for MSSA, showed higher activity against both MRSA strains. A period of 2 min of irradiation resulted in the complete destruction of MRSA 4524665 at a concentration of 10 ng/mL and MRSA 4529137 at a concentration of 16 ng/mL (Figure 7 and Figure 8). These differences may be attributed to variations in membrane properties between the strains, which could influence lectin binding and RB delivery efficiency.

The fact that the MRSA strains studied here, resistant to a broader spectrum of antibiotics, remained susceptible to RB-based photodynamic treatment emphasizes the potential of PACT as a resistance-independent antimicrobial strategy, since its mechanism relies on ROS generation rather than the biochemical inhibition pathways targeted by conventional antibiotics.

To summarize, these results confirm that the ConA-RB and WGA-RB conjugates are effective against susceptible Gram-positive and Gram-negative bacteria, as well as against clinical multidrug-resistant pathogens. The ability to achieve total bacterial eradication at low concentrations and low light doses highlights the clinical promise of these conjugates, especially in an era of escalating antimicrobial resistance.

## 3. Materials and Methods

### 3.1. Materials

Concanavalin A from *Canavalia ensiformis* (Jack bean) (ConA) was purchased from Sigma-Aldrich, St. Louis, MA, USA and wheat germ agglutinin (WGA) from wheat germ (*Triticum aestivum*) was purchased from Lectinotest, Lviv, Ukraine.

All reagents used in this study were of analytical grade: *N*-Hydroxysulfosuccinimide sodium salt (Sulfo-NHS)—Sigma-Aldrich, USA; *N*-ethyl-*N*′-(3-dimethylaminopropyl) carbodiimide (EDC)—Glentham Life Sciences Ltd., Corsham, UK; and Rose Bengal (RB)—Alfa Aesar, Morecambe, UK.

### 3.2. Bacterial Growth

Cultures of *Staphylococcus aureus* (ATCC 25923), *Escherichia coli* (ATCC 25922), *Pseudomonas aeruginosa* (ATCC 25668), and *Salmonella paratyphi* B (ATCC 8759) were purchased from ATCC (Manassas, VA, USA). The clinical strains MRSA 4524665 and MRSA 4529137 were kindly donated by Dr. Haim Ben-Zvi and Dr. Esti Michael (Rabin Medical Center, Petach Tikva, Israel). The bacteria were grown on Brain Heart Agar (BHA, Acumedia, Lansing, MI, USA) plates for 24 h, transferred to Brain Heart Broth (BH, Acumedia, Lansing, MI, USA), and incubated at 37 °C with shaking at 170 rpm until reaching OD_660_ ≈ 0.2. Bacterial suspensions were diluted to OD_660_ = 0.10 ± 0.02 (10^8^ CFU/mL) and further diluted to 10^4^ CFU/mL for experiments.

### 3.3. Agglutination Study

Bacterial cultures (*S. aureus*, *E. coli*, *P. aeruginosa*, and *S. paratyphi* B) were grown as described in Section 3.2 until reaching OD_660_ ≈ 0.2. ConA and WGA were dissolved in 0.1 M PBS, pH 7.4, to obtain a concentration of 1 mg/mL. For the bacteria agglutination activity test, 50 μL of each bacterial suspension was mixed with solutions of ConA or WGA (1 mg/mL each) to obtain a final volume of 100 μL on a clean glass slide. The mixtures were stirred with a sterile inoculation loop, and bacterial agglutinating activity was monitored using an optical microscope (Olympus CKX53, Tokyo, Japan) after 2 min. Untreated cells served as controls. The visible clumping of bacteria indicated agglutination, while a smooth homogeneous suspension meant a negative result.

### 3.4. Synthesis and Characterization of Lectin–RB Conjugates

The conjugates were synthesized following the method described by Cantelli et al. [40]. Rose Bengal disodium salt was dissolved in DMSO at a concentration of 10 mM. Solid EDC and sulfo-NHS were added under continuous stirring to achieve final concentrations of 27.5 mM and 15 mM, respectively. This solution was allowed to react for 5 h. The activated Rose Bengal was then mixed with ConA or WGA (0.05 mM each) in 0.1 M sodium carbonate buffer (pH 9.0) and incubated overnight under mild stirring conditions to ensure complete conjugation. The free dye was separated from the conjugate with Sephadex G 50 using 0.1 M sodium carbonate buffer (pH 9.0) as an eluent.

### 3.5. Determination of Rose Bengal to Lectin Ratios in the Conjugates

The concentration of RB in the conjugate was measured by dissolving it in 0.1 PBS, pH 7.4, assessing the absorbance at 547 nm, and comparing the results to a standard calibration curve of free RB. ConA and WGA concentrations in conjugates were determined using the Bradford method for protein quantification using individual lectins as standards for building calibration curves. The Rose Bengal/lectin ratio was calculated, taking into account the molecular weights of the components [58].

### 3.6. Antimicrobial Activity Test

The toxicity of the ConA-RB conjugate was evaluated in comparison to free RB. Bacterial suspensions containing 10⁴ CFU/mL of *S. aureus*, *E. coli*, *P. aeruginosa*, and *S. paratyphi* B were subjected to varying concentrations of free RB and lectin–RB conjugates. The mixtures were pre-incubated in a shaker at 100 rpm under dark conditions for 15 min for all bacteria. After pre-incubation, 0.1 mL of each solution at chosen time intervals was placed on Brain Heart Agar (BHA) and incubated overnight at 37 ± 1 °C in the dark. Bacterial colony-forming units (CFU) were counted using a Scan 500 colony counter (Interscience, Saint-Nom-la Bretèche, France).

The antimicrobial activity of the ConA-RB and WGA-RB conjugates was assessed under illumination and in the dark in a 48-well polystyrene clear flat bottom plate (Falcon^®^, Corning, AZ, USA) with 1 mL of conjugate solution in sterile saline at varying concentrations. Bacterial suspensions at 10^5^ CFU/mL (100 µL) were added to 900 µL of the conjugate solutions, yielding a final bacterial concentration of 10^4^ CFU/mL for all experiments. The mixtures were pre-incubated for 15 min for all the bacteria. After pre-incubation, the plates were either exposed to light under shaking at 100 rpm or kept in the dark as controls. During light exposure, 0.1 mL samples were taken at chosen time intervals and cultured on BHA overnight at 37 ± 1 °C in the dark, followed by CFU counting.

Light irradiation was performed using an 18 W LED lamp (OSRAM, model L18W/765, cool daylight, Munich, Germany). The lamp emitted light in the 400–800 nm range, with a fluence rate of 26 mW/cm^2^ and an intensity of 135 klux. Light intensity was measured using an LX-102 light meter (Lutron, Taipei, Taiwan). Samples were positioned at a distance of 8 cm from the lamp, and light doses of 3.1, 7.8, and 23.4 J/cm^2^ were applied for *S. aureus* and 46.8, 93.6, and 140.4 J/cm^2^ for Gram-negative bacteria.

### 3.7. Statistical Analysis

The results obtained from at least three independent experiments carried out in duplicates were analyzed by single-factor ANOVA analyses. The difference between the results was considered significant when the *p*-value was less than 0.05. Quantitative results are presented as the mean ± standard error.

## 4. Conclusions

This study demonstrates that conjugation of the photosensitizer Rose Bengal with the carbohydrate-binding lectins ConA and WGA significantly enhances the photodynamic antibacterial efficacy of the PS against both Gram-positive and Gram-negative bacteria. The synthesized conjugates allowed for the targeted delivery of RB to bacteria. Microscopic analysis demonstrated strong agglutination of bacterial cells with the lectins, supporting the role of the latter in targeting Gram-positive and Gram-negative cells. In the case of clinical strains, WGA-RB was more effective against the MRSA strains than the ConA-RB conjugate. For the other types of bacteria, photodynamic assays showed that the ConA-RB conjugate exhibited significantly higher antibacterial activity compared to both free RB and WGA-RB. WGA-RB also demonstrated more enhanced antibacterial activity than free RB, especially against Gram-positive bacteria. Among the Gram-negative strains tested, *E. coli* was the most susceptible to the treatment by both lectin-conjugated RB compounds, while *Salmonella paratyphi* B demonstrated the highest persistence against all treatments. Lectin–photosensitizer conjugates are promising antimicrobial photodynamic agents. By selecting the appropriate lectins for a specific type of bacteria, the effectiveness of PACT can be significantly increased. This conjugation strategy offers a potent antimicrobial treatment that may become an alternative to antibiotics, especially in the case of antibiotic-resistant strains.

## Data Availability

All data are available in the current publication.
